# *In-vivo* Dynamics of the Human Hippocampus across the Menstrual Cycle

**DOI:** 10.1038/srep32833

**Published:** 2016-10-07

**Authors:** Claudia Barth, Christopher J Steele, Karsten Mueller, Vivien P. Rekkas, Katrin Arélin, Andre Pampel, Inga Burmann, Jürgen Kratzsch, Arno Villringer, Julia Sacher

**Affiliations:** 1 Department of Neurology, Max Planck Institute for Human Cognitive and Brain Sciences, Leipzig, Germany; 2 Cerebral Imaging Centre, Douglas Mental Health Institute, Department of Psychiatry, McGill University, Montreal, Canada; 3 CAMH Research Imaging Centre and Campbell Family Mental Health Research Institute at the Centre for Addiction and Mental Health and the Department of Psychiatry, University of Toronto, Toronto, Canada; 4 Clinic of Cognitive Neurology, University of Leipzig, Leipzig, Germany; 5 Leipzig Research Center for Civilization Diseases, University of Leipzig, Germany; 6 Institute for Laboratory Medicine, Clinical Chemistry and Molecular Diagnostics, University Hospital Leipzig, Leipzig, Germany; 7 Integrated Research and Treatment Center Adiposity Diseases, University of Leipzig, Germany; 8 Berlin School of Mind and Brain, Mind and Brain Institute, Berlin, Germany

## Abstract

Sex hormones fluctuate during the menstrual cycle. Evidence from animal studies suggests similar subtle fluctuations in hippocampal structure, predominantly linked to estrogen. Hippocampal abnormalities have been observed in several neuropsychiatric pathologies with prominent sexual dimorphism. Yet, the potential impact of subtle sex-hormonal fluctuations on human hippocampal structure in health is unclear. We tested the feasibility of longitudinal neuroimaging in conjunction with rigorous menstrual cycle monitoring to evaluate potential changes in hippocampal microstructure associated with physiological sex-hormonal changes. Thirty longitudinal diffusion weighted imaging scans of a single healthy female subject were acquired across two full menstrual cycles. We calculated hippocampal fractional anisotropy (FA), a measure sensitive to changes in microstructural integrity, and investigated potential correlations with estrogen. We observed a significant positive correlation between FA values and estrogen in the hippocampus bilaterally, revealing a peak in FA closely paralleling ovulation. This exploratory, single-subject study demonstrates the feasibility of a longitudinal DWI scanning protocol across the menstrual cycle and is the first to link subtle endogenous hormonal fluctuations to changes in FA *in vivo*. In light of recent attempts to neurally phenotype single humans, our findings highlight menstrual cycle monitoring in parallel with highly sampled individual neuroimaging data to address fundamental questions about the dynamics of plasticity in the adult brain.

Accumulating evidence on the effects of estrogen outside of the reproductive system supports an influence of estrogen on brain structure and function^1^,^2^,^3^,^4^. Several rodent studies have shown strong effects of estrogen on adult neurogenesis^5^, electrophysiological and structural properties of the brain^6^, interactions with main neurotransmitter systems^7^, regulation of gene transcription^8^,^9^, and behavior^2^. Considering the prominent sexual dimorphism seen in many neuropsychiatric disorders, such as major depressive disorder^10^, endogenous sex hormones may play an essential role in modulating human brain states and mood. Epidemiologic studies report an increased risk for depressed mood when endogenous sex hormone levels fluctuate or decline rapidly from elevated levels, such as during the menopausal transition^11^,^12^ or early postpartum^13^. Changes in hippocampal function and morphology have been reported during depressive episodes in young adulthood^14^ and these findings have been replicated in different samples^15^,^16^. Given the high density of estrogen receptors in the hippocampus^17^, this region is of particular relevance for exploring whether subtle endogenous sex hormone fluctuations can induce neuroplastic change in humans.

Estrogen has been shown to elicit neurotrophic effects including promoting synaptogenesis and strengthening glia-structure in the rodent hippocampus^18^. In humans, the influence of estrogen on hippocampal morphology has been investigated by focusing on the changes that occur during puberty, when sex hormone levels rise substantially. During this period, increased estrogen levels have been shown to parallel greater volume in hippocampal regions^19^,^20^. Indirect support for estrogen-induced neuroplastic processes in the hippocampus stems from research in Turner syndrome, a condition of severe estrogen deficiency in females that is the result of a complete or partial absence of the X-chromosome^21^. Neuroimaging findings show reduced hippocampal grey and white matter in the presence of this condition^21^. Investigating the effect of endogenous estrogen on hippocampal neuroplasticity in humans *in vivo* is a relatively recent scientific endeavor. Most existing data are either derived from male-to-female comparisons^22^, cross-sectional studies^23^, or clinical populations^21^,^24^. Within-subject designs are challenging and therefore rare. Yet, such an approach may be more suitable to detect potential effects of subtle estrogen changes in the female brain.

The menstrual cycle provides a natural experimental set-up to investigate whether physiological sex hormonal fluctuations influence brain morphology. Most prior studies exploring the link between estrogen and female brain plasticity have focused on comparisons of women either on or off oral contraception^25^, sometimes reporting menstrual cycle effects as secondary findings^26^. Hormonal contraceptive use has been associated with increased grey matter in cortical regions^25^ and changes in cerebral white matter structure^27^. During the natural menstrual cycle, Protopopescu and colleagues found grey matter volume changes in the right anterior hippocampus in the high estrogen phase^28^. However, monitoring of the menstrual cycle in this study was based on self-reports, the authors did not assess sex hormone levels in saliva or blood, and the analysis was limited to two assessments across the menstrual cycle^28^. These caveats point to the most common challenge scientists face when trying to determine menstrual cycle phase, namely, self-reports are unreliable. It is estimated that only about 40% of women correctly report the time of their last menstrual bleeding when the time of recall was 3 weeks after bleeding cessation or longer^29^. More accurate monitoring methods include the assessment of body temperature and the detection of sex hormone concentration in blood and urine. However, these methods also have limitations, particularly when they are not applied in combination. Non-invasive daily recording of body temperature is highly influenced by day-to-day variability in the measurement procedure itself, cycle variability and the effects of illness, medication, diet, and fluctuations in sleeping patterns^30^. Blood sampling at a single time point to assess menstrual cycle phase is insufficient because the menstrual cycle is characterized by high estrogen levels before ovulation (follicular phase) and has a second blunted peak after ovulation and before the onset of menses (luteal phase). Multiple blood sampling time points are thus needed for accurate menstrual cycle monitoring. In addition, confirming ovulation, for instance by assessing lutropin (LH) levels in urine, seems advisable given that an anovulatory cycle can randomly occur in women with otherwise good menstrual cycle health^31^.

When accurate menstrual cycle tracking is performed, the resulting analysis of ovarian hormone fluctuation and brain structure often yields interesting data: several rodent studies have shown that estrogen has neurotrophic and neuroprotective effects on grey and white matter structure across the estrous cycle^32^,^33^.

So far, there is no direct method available to quantify white matter microstructural changes in the human brain *in vivo*. However, it is possible to assess parameters that are sensitive to alterations in white matter microstructure. Diffusion weighted imaging (DWI) is a MRI technique that measures the directionality of molecular water diffusion^34^ and enables non-invasive observation of structural reorganization in the human brain^35^. As axons provide natural barriers for water diffusion, the diffusion profile within a certain brain region can be used to infer local structural properties^35^. This method has been used successfully to detect white matter changes characteristic of axonal damage and demyelination^34^ and reorganization associated with functional recovery after brain injury^36^. Scalar measures of DWI include fractional anisotropy (FA, the most frequently used metric of the diffusion tensor), which quantifies how strongly directional the local tissue is^34^. A decrease in FA reflects decreased anisotropic diffusion that can indicate white matter damage^37^,^38^. Yet, FA is also sensitive to microstructural changes in fiber density and myelination^39^. Axial diffusion along the primary axis and radial diffusion perpendicular to the primary diffusion axis can be investigated within each FA cluster to specify diffusion directionality in more detail^40^,^41^. Both diffusion properties have been associated with different biological underpinnings: radial diffusion (RD) with myelin changes^42^ and axial diffusion (AD) with axonal damage^34^. Understanding the relative contribution of these parameters to FA values allows for a more in depth interpretation^40^,^41^ of potential estrogen effects on human white structure microstructure across the menstrual cycle.

In this study, we aim to explore the influence of endogenous estrogen on hippocampal white matter measures. Investigating the influence of subtle physiological estrogen fluctuations on structural connectivity in health can reflect on the capacity of the human brain to adapt to the environment. In contrast, treatment and scar effects can bias data obtained from cross-sectional studies in clinical populations. The present work, a heavily sampled longitudinal dataset with detailed information on hormonal status, could prove a powerful means to detect subtle brain changes in synchrony with the menstrual cycle.

## Materials and Methods

### Participant

We acquired 30 longitudinal scans of a single, 32-year old, Caucasian female with a documented history of regular menstrual cycles (mean = 28 ± 1 day). The subject was physically healthy, with no history of any psychiatric, neurological, or chronic illnesses. The subject was right-handed, of normal weight (BMI 20.2), and free of medication, including no use of contraceptives or antidepressants (i.e., antidepressant-naïve). She was a non-smoker, with no history of alcohol use, drug abuse, pregnancy, or period of breast-feeding. The participant was screened using the structured clinical interview for DSM to rule out any Axis I major mental disorders^43^ and Axis II personality disorders^44^. Any potential subclinical manic, depressive and anxiety symptoms were excluded by administration of the Hamilton Rating Scale for Depression (HAM-D)^45^, the Mood Spectrum self-report (MOODS-SR), and the State-Trait anxiety inventory (STAI). The subject was recruited through the institute’s volunteer database and provided written informed consent to participate. The study protocol was approved by the local ethics board of the University of Leipzig (EK-No.: 246-2009-09112009) and carried out in accordance with the Declaration of Helsinki.

### Hormone assays

We collected fasting blood samples to determine serum hormone levels of estrogen (pmol/l), progesterone (nmol/l), and LH (U/l) immediately prior to every MRI scan. Blood samples were analyzed at the Institute for Laboratory Medicine of the University Hospital Leipzig by the fully automated Roche Cobas® system (Roche, Basel, Switzerland). All samples were measured within one plate: intra-assay coefficients of variance (ICV) were within 3.2–6% for estrogen, 2.3–5.2% for progesterone, and 1.6–2% for LH. Ovulation was further confirmed by detecting LH surge, using urine ovulation tests (Diagnostik Nord GmbH hLH-K20 hLH Kassettentest).

### MRI data acquisition

**Figure 1:**
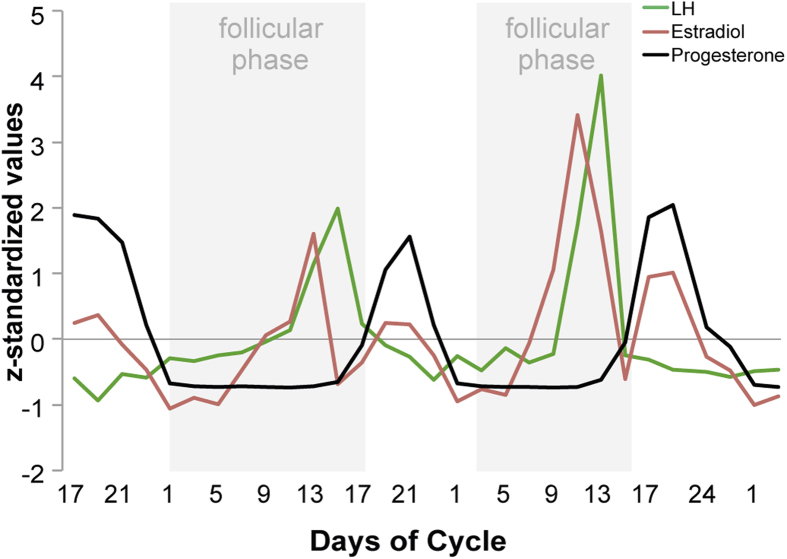
Endogenous ovarian sex hormone fluctuation. Characteristic patterns of serum estradiol (red line), progesterone (black line) and LH (green line) levels are displayed across two menstrual cycles (follicular phase highlighted in grey). The depicted data represent days of cycle with corresponding z-standardized, single hormone values, matching the single scan time points in chronological order. As expected, estradiol shows a first prominent peak in the periovulatory phase, followed by a second smaller peak in the late luteal phase. LH surges after the peak of estrogen, shortly before ovulation. Progesterone levels are low during the follicular phase and high during the luteal phase.

**Figure 2:**
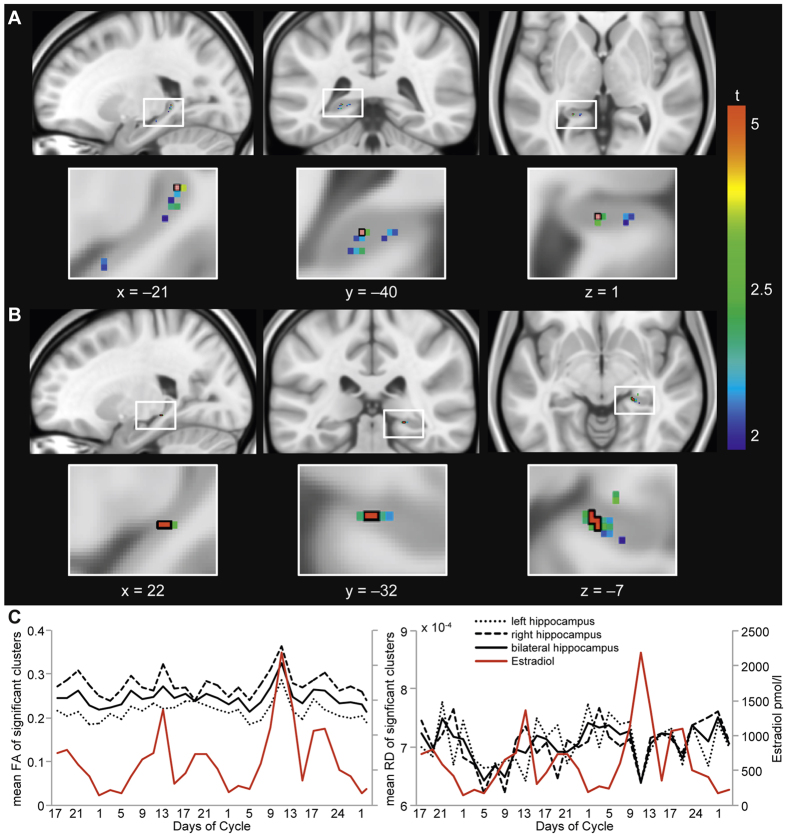
Estrogen-modulated FA differences and time course across the menstrual cycle in the bilateral hippocampus. Threshold-Free Cluster Enhancement (TFCE) voxel-wise FA correlation with respective estrogen levels in hippocampal ROI-masks (left (panel **A**) and right (panel **B**) hippocampus) are displayed. Red voxels outlined with black, superimposed on respective t-values, correspond to significant FWE-corrected results (p < 0.05). In panel **C**, FA (left) and RD (right) values from significant clusters, respectively peak voxel, are extracted and plotted versus estrogen levels (in red) across the menstrual cycles assessed.

MRI images were acquired every second or third day in two separate scanning sessions to cover two full menstrual cycles. Data from scanning session one was collected from January 6, 2012, to February 3, 2012 (16 scans in total) starting with cycle day 15 in the first cycle; to cycle day 21 in the second cycle. Therefore the first scan session bridges two menstrual cycles, covering one full menstrual cycle. The second scanning session spanned from October 31, 2012 to November 30, 2012 (16 scans in total) starting with cycle day 25 until cycle day 3. The time course of scanning according to the subject’s menstrual cycle is depicted in chronological order in Figures 1 and 2(C). Scanning sessions began on a different day of the menstrual cycle and were acquired to different seasons in order to control for potential scanner-drift artifacts and seasonal effects, respectively. Further, every scan was collected at the same time of the day (7.30 am) to control for circadian rhythm effects. Two scans were excluded: one because of missing hormone data and one because of a technical problem with DWI acquisition.

All images were acquired on a 3-Tesla Magnetom Verio scanner (Siemens, Erlangen, Germany) equipped with a 32-channel head coil. During each scanning session, diffusion data were acquired using a whole-brain, gradient-echo echo-planar imaging (EPI) sequence. The following parameters were used: 72 slices, slice thickness = 1.7 mm, no intersection gap, field of view = 220 mm^2^, TR = 13.8 s, TE = 100 ms, A/P phase encoding direction, 67 diffusion directions, transversal orientation, fat saturation, *b *= 0, 1000 s/mm^2^, bandwidth = 1346 Hz/pixel, GRAPPA parallel imaging and acquisition matrix = 128 × 128. The reconstruction matrix was the same as the acquisition matrix, and voxels were 1.7 mm isotropic. The total DWI scanning time was 16 minutes.

In addition, high-resolution T1-weighted images to test for grey matter structural changes were acquired using a whole-brain, three-dimensional Magnetization-Prepared Rapid Gradient Echo (MPRAGE) sequence. The Alzheimer’s Disease Neuroimaging Initiative (ADNI) standard protocol was used with the following parameters: TI = 900 ms, TR = 2300 ms, TE = 2.98 ms, flip angle = 9°, A/P phase encoding direction, bandwidth = 238 Hz/pixel, image matrix = 256 × 240, 176 partitions, field of view = 256 × 240 × 176 mm^3^, sagittal orientation, no fat suppression and average voxel size 1 × 1 × 1 mm^3^. The acquisition time was 9 minutes.

### Diffusion data analysis

Diffusion data were analyzed with FSL version 5.0.8 using the FMRIB’s software library (www.FMRIb.ox.ac.uk/fsl). Briefly, the following standard processing pipeline was applied before creating voxel wise maps of diffusion parameters: (1) eddy current correction to correct for gradient-coil distortions and small head motions, (2) removal of non-brain tissue using the Brain Extraction Tool (fractional threshold = 0.25), and (3) local fitting of the diffusion tensor at each voxel using DTIfit (FMRIB’s Diffusion Toolbox v2.0 (FDT)^46^).

Next, FA images were processed with FSL’s diffusion toolkit^47^,^48^. First, all images were nonlinearly aligned to the most representative FA image out of all images and transformed into 1 × 1 × 1 mm MNI152 standard space. Volumetric FA images were minimally smoothed (σ = 1 mm) and subsequently used in permutation-based nonparametric statistical analyses. The nonlinear warp derived from FA co-registration was also used to align the volumetric images of axial diffusivity (AD) and radial diffusivity (RD). This approach was used to examine the relative contribution of AD and RD to potential hormone-dependent changes in FA values *post hoc*.

### Structural data analysis

MPRAGE structural data was analyzed with FSL-VBM^49^ (http://fsl.fmrib.ox.ac.uk/fsl/fslwiki/FSLVBM), an optimized voxel-based morphometry (VBM) protocol^50^ carried out with FSL version 5.0.8. First, structural images were reoriented, brain-extracted and grey matter-segmented before being registered to the MNI 152 standard space using non-linear registration (www.fmrib.ox.ac.uk/analysis/techrep). The resulting images were averaged and flipped along the x-axis to create a left-right symmetric, study-specific grey matter template. Second, all native grey matter images were non-linearly registered to this study-specific template and “modulated” to correct for local expansion (or contraction) due to the non-linear component of the spatial transformation. The modulated grey matter images were then smoothed with an isotropic Gaussian kernel with a sigma of 3 mm. Finally, voxel wise general linear model was applied using permutation-based non-parametric testing, correcting for multiple comparisons across space.

### Statistical analysis

Based on previous work linking the menstrual cycle to grey matter changes and changes in functional connectivity^51^ in the hippocampus, we chose this structure as our region of interest (ROI). Hand drawn ROIs for right and left hippocampal white matter were derived from the thresholded (FA > 0.2) mean FA image and hippocampal segmentation of the Harvard-Oxford Subcortical Structural Atlas^52^ implemented in FSLView (FSL)^47^. To validate our hand drawn mask, we also generated intensity-based, atlas-defined anatomical masks of the bilateral hippocampus by using the Harvard Oxford Subcortical Structural Atlas, which were thresholded at 0.2 to account for white matter.

We correlated volumetric FA from the bilateral hippocampal ROIs with respective hormone levels using a nonparametric permutation-based approach (5000 permutations). We set the statistical threshold at a voxel-level at p < 0.05, after family-wise error (FWE) correction for multiple comparisons using threshold-free cluster enhancement^53^. The same approach was used for the structural data using anatomical atlas-defined hippocampal masks from the Harvard Oxford Subcortical Structural Atlas.

Regions identified in the previous step were subsequently used as masks to extract FA, AD and RD values for plotting and for calculating partial correlations with respective hormone values using SPSS Statistics 22 (p < 0.05). These analyses were corrected for set of scan sessions as a covariate of no interest, as both sessions were acquired 9 months apart.

## Results

### Hormone assessment

Hormone analyses confirmed typical patterns for estrogen, progesterone and LH levels characteristic of the ovulatory, follicular, and luteal phases of the menstrual cycle, across all cycles in the study. A plot of the measured hormone fluctuation across the menstrual cycles can be found in Fig. 1.

### Correlations within region of interest

To determine hippocampal microstructural differences related to specific endogenous hormone fluctuations during the menstrual cycle, volumetric FA values in the bilateral hippocampal ROIs were correlated with respective hormone levels. Estrogen levels were significantly correlated with volumetric FA in the bilateral hippocampus (right peak voxel: 22 −32 −7, *t* = 5.75 *p* = 0.0016; left peak voxel: −21 −40 1, *t* = 5.36 *p* = 0.0232; Fig. 2A,B). No such correlation was found for progesterone.

**Figure 3:**
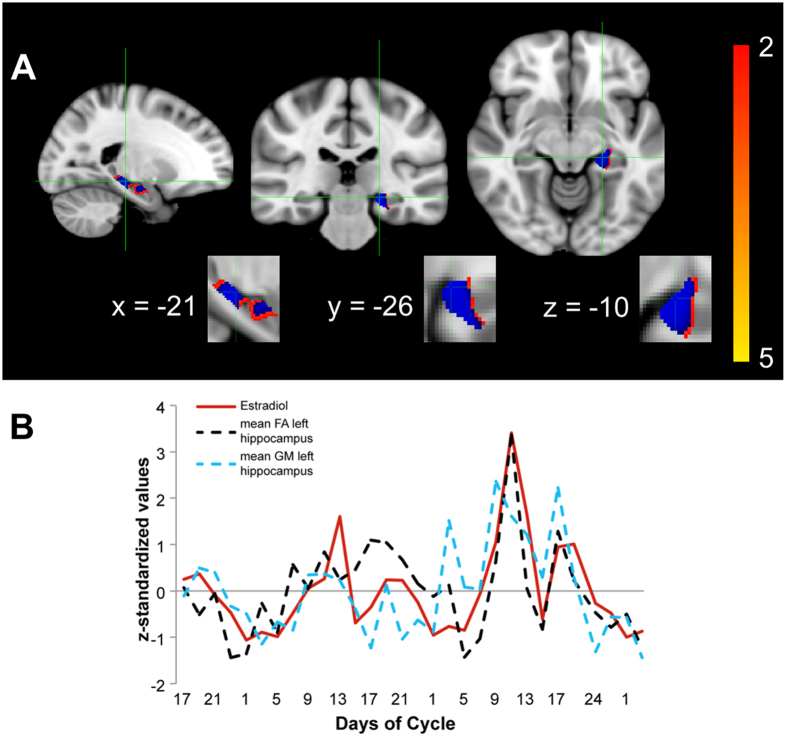
Estrogen-modulated grey matter differences in the left hippocampus. Threshold-Free Cluster Enhancement (TFCE) voxel-wise grey matter correlation with respective estrogen levels in hippocampal ROI-mask (hair cross on peak voxel; panel **A**: grey matter changes in left hippocampus) are displayed. Blue voxels, superimposed on respective t-values, correspond to significant FWE-corrected results (p < 0.05). In panel **B**, bilateral FA and left grey matter values from significant clusters, respectively peak voxel, are extracted and plotted versus estrogen levels (in red) across the menstrual cycles assessed.

To assess menstrual cycle-related hippocampal grey matter density changes, we performed a permutation-based nonparametric statistical analysis using FSL-VBM^49^. We found a significant correlation of estrogen levels with grey matter density in the left hippocampus (peak voxel: -20 -28 -8, t = 3.86, p = 0.0118; Fig. 3A), but not in the right hippocampus. To visualize hippocampal grey matter density changes in comparison to hippocampal FA changes across the menstrual cycle, we extracted mean grey matter values of the left hippocampus and plotted it against estrogen level and mean FA values of the left hippocampus (Fig. 3B, for details on how to access this dataset, see supplementary information).

### Correlations with region of interest extractions

To further assess the relationship between measures of diffusion in the hippocampus and estrogen levels across the menstrual cycle, we extracted mean FA, AD and RD values from significant voxels identified in the previous volumetric FA analysis in the hippocampal ROIs. To visualize scalar measure differences across the menstrual cycles, Fig. 2C includes plots of extracted hippocampal FA and RD values against menstrual cycle days. Estrogen levels were inversely correlated with RD (mean RD, *r *= −0.460, *p *= 0.012; Fig. 2C, bottom right) but not with AD (mean AD, *r *= 0.116, *p *= 0.547; not shown).

To evaluate a potential time lag in the effects of estrogen on white matter microstructure, we conducted a cross correlation analysis between estrogen levels and FA values to model the relationship of two time series and to display lag-associations at single points in time. This analysis revealed that the largest correlation between FA and estrogen occurred at zero time lag.

## Discussion

The current study provides evidence that hippocampal changes parallel fluctuations in endogenous ovarian hormone levels across the menstrual cycle. Specifically, greater FA was significantly associated with increased estrogen levels in bilateral hippocampus. Further, estrogen levels were negatively correlated with RD levels. These diffusion scalar changes in the hippocampus were extended by grey matter signal changes in one hippocampal hemisphere. These multimodal findings suggest significant dynamics in hippocampal structure across the menstrual cycle with a potentially myelin-related process underlying the white matter change.

Our results imply a rapid timeline for menstrual cycle associated structural hippocampal changes in the human brain. The peaks in FA in our data mirror the increases in estrogen, and are most prominent during the estrogen surge shortly before ovulation (Fig. 3, panel C). We found zero time lag in the cross correlation between FA values and estrogen levels, which suggests an immediate effect for high estrogen within a range of 2-3 days. This pattern is consistent with previous grey matter imaging findings that report an acute increase in hippocampal volume from the early follicular phase to the late follicular phase^54^, as well as the late luteal phase to the late follicular phase in humans^28^. Evidence from rodent studies also supports a remarkable degree of hippocampal plasticity change in rapid response to estrogen-rises across the estrous cycle^6^,^18^.

Several different processes could induce the changes in hippocampal FA that we report here. Since FA quantifies the strength of directionality in local tissue structure^34^, higher FA values in the hippocampus might point towards a higher directionality in hippocampal white matter tissue. On a cellular level, changes in white matter structure can be related to alterations in fiber organization, myelination, and changes in astrocytes and angiogenesis^55^. A potential mechanism that has been proposed to underlie white matter changes following learning-experiments is myelination^35^. In the current study, the increase in FA is primarily driven by a decrease in RD, a scalar measure that has been associated with myelin changes^42^. In humans, radial diffusivity has been proposed as a surrogate marker for myelin and radial diffusivity measures have successfully been used to characterize the extent of demyelination following traumatic brain injury^56^. Hence, the increased hippocampal FA and decreased RD associated with high estrogen levels, could reflect microstructural changes (e.g., such as myelination) due to the trophic effects of estrogen on white matter.

We stress that we do not provide any conclusive measurement for the specific biological microstructure that underlies the short-term dynamics of hippocampal changes we observe across the menstrual cycle. However, we have chosen a well-established FA threshold for identifying white matter^3^,^24^,^27^,^57^ within our hand-drawn hippocampal masks. White matter structure also seems to mediate the relationship between other steroid hormones, such as cortisol, and the hippocampus associated with longitudinal healthy ageing^58^. This may be a speculative explanation for the stronger association we observe between the steroid hormone estrogen and the diffusion scalar measures more closely associated with white matter structure (FA, RD) compared to both a diffusion scalar measure that has been described as an inverse measure of membrane density, that is both very similar in grey and white matter (MD = mean diffusivity) or the GM findings from our VBM analysis that are limited to one hemisphere (Fig. 3). However, although informative, diffusion-weighted MRI is an indirect measure of structural changes in the brain and the underlying cellular mechanisms remain to be clarified. The hippocampus represents a heterogeneous brain region and both, grey matter volume^59^ and white matter structure^58^ have been linked to hippocampal neuroplastic changes in health. The hippocampal changes we find across the menstrual cycle seem to be located at grey-white matter boundaries (Fig. 2) and we cannot exclude that grey matter changes or changes in vasculature contribute to the changes we observe in diffusion scalar measures.

Combining high-resolution human MRI data with histologically validated neuroimaging datasets in rodents can be a useful approach to investigate the changes that occur at the level of the neuronal microstructure. A pioneering study by Sagi and colleagues (2012) compared DWI imaging in humans, with combined DWI imaging/histological analysis in rodents, after each group completed a comparable short-term memory task^60^. The authors found a significant change in FA, and mean diffusivity, in hippocampal regions 2 hours post-learning in both the human and rodent data. The histological results from the rat study revealed an increase in synaptophysin and brain-derived neurotrophic factor (BDNF), suggesting a structural remodeling process following the learning task. BDNF is involved in long-term potentiation (LTP)^60^ which is believed to represent a cellular correlate of learning and memory^61^. Estrogen has been found to increase BDNF expression in the hippocampus^62^ and an interaction between estrogen and BDNF has been proposed to mediate women’s risk for pathologies characterized by memory-loss^63^. A recent study reports a positive correlation between BDNF levels in blood serum and hippocampal volume across the menstrual cycle^54^. Considering these points, one could speculate that estrogen dependent FA changes in the hippocampus across the menstrual cycle could reflect dynamic structural changes comparable to the neuroplasticity changes that have been observed following short-term learning. A multi-modal study-design across rodents and humans could be applied to test this hypothesis in the future.

One limitation of our current study is that we did not include a sequence to specifically quantify potential hormone-related water shifts in the brain that could also account for the FA change. While we acknowledge the possibility that menstrual-cycle related extracellular/intracellular water shifts may contribute to our findings, we consider this unlikely to be the driving mechanism for the following reasons: (1) A substantial change in hydration-status by a strict hydrating/thirsting protocol that has been shown to induce a significant enlargement of ventricular structure and an increase in cerebrospinal fluid (CSF) did not show any significant grey or white matter changes within the hippocampal region^64^. (2) We did not find any significant results when analyzing CSF changes and estrogen levels across the menstrual cycle in our dataset. (3) The short-time water changes that have been described to be influenced by fluctuating endogenous sex hormones previously^65^ show a correlation with progesterone, not estrogen. We did not find any significant correlation between progesterone and any of the scalar diffusion measures. In summary, these observations from the literature and our own data support that a shift from extracellular/intracellular water is unlikely to explain the estrogen-associated hippocampal changes we find across the menstrual cycle. We also acknowledge that we did not include any neurocognitive testing in our protocol. Thus, we cannot address whether the potential changes in white matter across the menstrual cycle may relate to behavioral changes. However, grey matter changes across the menstrual cycle were not associated with any significant changes in cognitive abilities in healthy subjects^54^. Detailed cognitive assessment may be more relevant to clinical populations, such as patients with premenstrual dysphoric disorder. A major aim of our study was to test the feasibility of a longitudinal DWI design across the female menstrual cycle.

In summary, this study is the first to link subtle hormonal fluctuations occurring during the menstrual cycle to changes in FA. Our findings indicate a remarkable degree of hippocampal plasticity in response to estrogen on a very rapid timescale–within days. These data are consistent with evidence from other studies investigating the impact of the menstrual cycle on the human brain^28^,^54^,^65^,^66^. A noteworthy consistency of neuroplasticity changes during the ovulatory phase, when estrogen levels peak, prevails across study-design, subject- and scan-number. Our study introduces an approach for simultaneously mapping longitudinal characteristics in endogenous hormone and hippocampal dynamics. We acknowledge the limitations from this single subject design. In light of recent attempts to neurally phenotype single humans^67^,^68^, our findings highlight menstrual cycle monitoring in parallel with highly sampled individual neuroimaging data to address fundamental questions about the dynamics of plasticity in the adult brain. Similar protocols might be necessary to adequately capture plasticity dynamics when designing future MRI studies that include female participants. The present study represents an important first step towards creating a personalized map of the individual human brain by integrating potential mediators, such as menstrual cycle phase.

## Additional Information

**How to cite this article**: Barth, C. *et al*. *In-vivo* Dynamics of the Human Hippocampus across the Menstrual Cycle. *Sci. Rep*. **6**, 32833; doi: 10.1038/srep32833 (2016).

## Supplementary Material

Supplementary Information
