# Supplementary material for: In-vivo Dynamics of the Human Hippocampus across the Menstrual Cycle
Source: Sci Rep. 2016 Oct 7;6:32833. doi: 10.1038/srep32833 (PMC5054394; doi:10.1038/srep32833)
Supplement: Supplementary Information [file srep32833-s1.pdf]

In-vivo Dynamics of the Human Hippocampus across the Menstrual Cycle

Claudia Barth[1*], Christopher J Steele[1,2], Karsten Mueller[1], Vivien P Rekkas[3], Katrin Arelin[1,4,5], Andre Pampel[1], Inga Burmann[1], Jürgen Kratzsch[6], Arno Villringer[1,4,5,7,8] , Julia Sacher[1,4].

[1] Department of Neurology, Max Planck Institute for Human Cognitive and Brain Sciences, Leipzig, Germany;[2] Cerebral Imaging Centre, Douglas Mental Health Institute, Department of Psychiatry, McGill University, Montreal, Canada;[3] CAMH Research Imaging Centre and Campbell Family Mental Health Research Institute at the Centre for Addiction and Mental Health and the Department of Psychiatry, University of Toronto, Toronto, Canada; [4] Clinic of Cognitive Neurology, University of Leipzig, Leipzig, Germany;[5] Leipzig Research Center for Civilization Diseases, University of Leipzig, Germany;[6] Institute for Laboratory Medicine, Clinical Chemistry and Molecular Diagnostics, University Hospital Leipzig, Leipzig, Germany;[7] Integrated Research and Treatment Center Adiposity Diseases, University of Leipzig, Germany;[8] Berlin School of Mind and Brain, Mind and Brain Institute, Berlin, Germany.

S1 Overview of Data made openly available according to the Committee on Best Practice in Data Analysis and Sharing (COBIDAS) guidelines as published by the Organization for Human Brain Mapping (http://biorxiv.org/content/early/2016/05/20/054262).

Aspect	Notes
**Shared material with corresponding data format**	Unthresholded t- and corrected p-maps of DWI data (bilateral hippocampus, contrast 1 for estrogen; corresponding to Figure 2)<br>- **Hipp_L_estrogen_DWI_tfce_corrp_tstat1_78014.nii.gz**<br>- **Hipp_L_estrogen_DWI_tstat1_78014.nii.gz**<br>- **Hipp_R_estrogen_DWI_tfce_corrp_tstat1_78014.nii.gz**<br>- **Hipp_R_estrogen_DWI_tstat1_78014.nii.gz**
	Unthresholded t- and corrected p-maps of VBM data (bilateral hippocampus, contrast 1 for estrogen; corresponding to Figure 3)<br>- **Hipp_L_estrogen_VBM_tfce_corrp_tstat1_78014.nii.gz**<br>- **Hipp_L_estrogen_VBM_tstat1_78014.nii.gz**<br>- **Hipp_R_estrogen_VBM_tfce_corrp_tstat1_78014.nii.gz**<br>- **Hipp_R_estrogen_VBM_tstat1_78014.nii.gz**
**URL, access information**	Neurovault: public repository providing open access to functional and structural data on the human brain: **http://neurovault.org/collections/1543/**
**Ethics compliance**	Study and recruitment procedures were carried out in accordance with the Declaration of Helsinki and approved by the research ethics board of the University of Leipzig (EK-No.: 246—2009—09112009).

*[1] VBM = voxel-based morphometry, *[2] DWI = diffusion-weighted imaging
